# Use of donor human milk in nonhospitalized infants: An infant growth study

**DOI:** 10.1111/mcn.13128

**Published:** 2021-01-06

**Authors:** Solange Bramer, Robert Boyle, Gillian Weaver, Natalie Shenker

**Affiliations:** ^1^ Imperial College London Medical School St Mary's Hospital London UK; ^2^ Department of Paediatrics Imperial College London, St Mary's Hospital London UK; ^3^ The Human Milk Foundation, Daniel Hall Building Rothamsted Institute Herts UK; ^4^ Department of Surgery and Cancer Imperial College London London UK

**Keywords:** breastfeeding, feeding problems, growth, human milk, infant feeding, infant growth, milk banks

## Abstract

When mother's own milk (MOM) is unavailable or insufficient, donor human milk (DHM) is recommended as the next best alternative for low birthweight infants. DHM use for healthy, term infants is increasing, but evidence for growth and tolerability is limited. This retrospective study evaluated growth in term infants in the community who received DHM from a UK milk bank. Mothers of infants receiving DHM between 2017 and 2019 were contacted (*n* = 49), and 31 (63.2%) agreed to participate. Fourteen infants received DHM as a supplement to other feeds (MOM and/or infant formula) and 17 were exclusively fed DHM where breastfeeding was impossible (range: 3–6 weeks). Growth was assessed by deriving *z*‐scores using the WHO standard for infant growth and compared with 200 exclusively breastfed infants. Multivariate regression analysis revealed no feeding method‐specific association between *z*‐score and age, nor between weight and age, suggesting that *z*‐scores and growth velocity were not affected by feeding exclusive MOM, supplemental DHM or exclusive DHM. DHM was well‐tolerated with no adverse events that led to early cessation. After receiving supplemental DHM group, 63% of infants whose mothers had no physical barrier to breastfeeding (5/8 infants) were exclusively breastfed. This novel study reports adequate growth outcomes of healthy nonhospitalized infants receiving DHM, either as the sole milk source or supplement. Prospective studies are needed to confirm whether DHM is a suitable feeding alternative for term infants in the community, optimal durations, as well as the impact of DHM availability on breastfeeding rates and maternal mental health.

## Abstract 2

DHM recipients were compared with the who infant growth standard (World Health Organization, 2020) by deriving *z*‐scores. The WHO Anthro Macro (World Health Organization, 2011 ) was used to calculate weight‐for‐age, length‐for‐age and head circumference‐for‐age *z*‐scores; *z*‐scores are the standard deviation of an infant's anthropometric measurements when compared with the WHO standard for infant growth and are gender and age specific.

**Key messages**

- This study retrospectively investigated the growth of nonhospitalized infants who received donor human milk (DHM) in the community exclusively or as a supplement to their regular feeds.
- A multivariate regression analysis revealed neither a feeding method‐specific association between *z*‐score and age nor a feeding method‐specific effect on growth velocity.
- To our knowledge, this is the first study to analyse the growth of healthy infants in the community fed DHM.
- Future studies should aim to validate these findings and explore the impact on breastfeeding rates and maternal mental health in larger populations.

## 1 INTRODUCTION

The World Health Organization (WHO) recommends that infants should be exclusively breastfed for 6 months, but this target is only met in 38% of infants worldwide (Victora et al., 2016). When mother's own milk (MOM) is unavailable or of insufficient quantity to meet nutritional needs, donor human milk (DHM) is recommended by the WHO as the safest alternative to maternal milk. The use of DHM has been limited to vulnerable groups since the 1980s primarily on the basis of rationing and cost‐effectiveness and is particularly used when maternal milk supply is insufficient for preterm infants. DHM use is largely restricted to preterm infants based on gestational age cut‐offs (typically <32 weeks gestation at birth) and weight <1500 g. Most research has focused on these infant populations, as a result of a reduced risk of necrotizing enterocolitis (M. Quigley, Embleton, & McGuire, 2019). Some studies have raised concerns about a negative impact on growth (Chowning et al., 2016; Colaizy, Carlson, Saftlas, & Morriss, 2012; Corpeleijn et al., 2016; Madore et al., 2017), although a recent Cochrane review found no difference in growth at 1 year between infant formula (IF)‐ and DHM‐fed preterm and low‐birth‐weight infants (M. Quigley, Embleton, & McGuire, 2018).

Qualitative research suggests mothers may perceive DHM as a bridge to overcome initial breastfeeding difficulties (Kair & Flaherman, 2017), rather than as a failure on their part when formula is used. When used in the context of optimal lactation support, DHM use in neonatal intensive care unit (NICU) can support maternal breastfeeding (Adhisivam et al., 2017; Kantorowska et al., 2016; Wilson et al., 2018), whereas the early introduction of IF is correlated with early breastfeeding cessation (Chantry, Dewey, Peerson, Wagner, & Nommsen‐Rivers, 2014; McCoy & Heggie, 2020). Two randomized controlled trials evaluated early, controlled supplementation with IF or DHM for newborns with significant weight loss, finding neither form of supplementation‐influenced breastfeeding duration (Flaherman, Cabana, McCulloch, & Paul, 2019; Kair, Flaherman, & Colaizy, 2019).

Broader availability and provision of DHM may therefore be a tool to support maternal breastfeeding, but its effect on the growth and feeding outcomes of populations of healthy, full‐term babies needs consideration. As a consequence of the limited availability of DHM, no data exist on the growth of nonhospitalized term infants fed with DHM or the experiences of their caregivers. This study evaluated whether DHM exclusively, or as a feeding supplement, would support the growth of infants at home. Feeding tolerance and exclusive breastfeeding rates after receiving DHM were also investigated.

## 2 METHODS

### 2.1 Participants

This retrospective observational study evaluated feeding and growth outcomes in term infants who received DHM in the community from a single milk bank in England. The Hearts Milk Bank (HMB) supplies pasteurized DHM to neonatal units in England and Wales in accordance with the National Institute for Health and Care Excellence Clinical Guideline 93 for the operation of a human milk bank (National Institute for Health and Care Excellence, 2010). Surplus pasteurized DHM is provided on clinical request to infants in the community who are not exclusively receiving MOM.

The study's three cohorts were based on the infants' feed type: exclusive MOM, exclusive DHM and supplemental DHM. Infants in the exclusive DHM group had received DHM as their sole type of feeding for at least 3 weeks. Mothers of infants receiving exclusive DHM were completely unable to breastfeed. The supplemental DHM group consisted of infants who had received DHM from the HMB to supplement their regular feeding, which was MOM, IF, or a combination of both. These mothers also received support for lactation difficulties from the HMB. The MOM cohort consisted of children exclusively fed MOM and whose mothers had donated their milk to the HMB. Exclusion criteria for the MOM group were death of the infant, missing infant growth data and incomplete information about maternal demographics.

### 2.2 Data collection and procedures

The primary outcome was the growth of infants receiving DHM. For the MOM group, weight at birth and at the time of their mother's recruitment to become a milk donor were extracted from the HMB donor screening questionnaire. Parents of the infants in the two DHM recipient groups could choose to have a telephone interview or answer an online survey (available at: https://imperial.eu.qualtrics.com/jfe/form/SV_cOSJP7ssU3uRTgN). The weight measurements of the MOM cohort were self‐reported by their mothers on the donor screening questionnaire. Weight, length and head circumference measurements were collected DHM recipients from the infants' personal child health records. These are not medical records but rather baby books that parents keep containing their infant's vital health information. Healthcare professionals will record the child's growth in this book. All growth measurements recorded for each individual infant were extracted, even if these measurements were taken before the baby had started or after the baby had stopped receiving DHM feeds. Clinical and demographic data about the infants and their mothers were also collected for all the infants. The HMB operates according to the National Institute for Health and Care Excellence guideline 93, which states that the use of DHM must be tracked and records kept for 30 years. Records are therefore kept of the volume of DHM provided to each family, and these were used to calculate the volume of DHM received by each baby.

The growth analysis consisted of two parts as the growth of infants receiving DHM was compared with both the WHO standard for infant growth and the reference feeding group (MOM recipients). DHM recipients were compared with the WHO infant growth standard (World Health Organization, 2020) by deriving *z*‐scores. The WHO Anthro Macro (World Health Organization, 2011) was used to calculate weight‐for‐age, length‐for‐age and head circumference‐for‐age *z*‐scores; *z*‐scores are the standard deviation of an infant's anthropometric measurements when compared with the WHO standard for infant growth and are gender and age specific. WHO growth charts are based on infants born at term (>37 weeks gestation), and therefore, preterm infants were excluded from this analysis. A *z*‐score of 0 indicates that the infant is on the 50th centile for that measurement. To compare the DHM recipients to the MOM recipients, growth velocity normalized to the infant's birth weight (weight gain per day, g/kg/day) was calculated for each infant in the study. All infants were included in this analysis. Subgroup analyses were performed within each feeding group for term infants. Multivariate regression models were created to evaluate the effect of feeding method (exclusive DHM, supplemental DHM and exclusive MOM) on *z*‐scores and growth velocity.

Feeding tolerance was investigated in DHM recipients during the telephone interviews and online survey. One study author was responsible for the data collection. Mothers were asked whether they noticed changes to their baby's behaviour or feeding pattern since using DHM. They were also asked binary questions about the incidence of the following symptoms: colic, fussiness while feeding, cramping, regurgitations, wheezing and skin rashes. This symptom assessment was based on guidelines created for randomized clinical trials that evaluate new IFs (Eskander & Nguyen, 2019). The incidence of adverse reactions to the DHM, along with the reasons for stopping DHM feeds, was also investigated, to evaluate whether feeding tolerance had played a part. Breastfeeding outcomes were only studied in the supplemental DHM group as exclusive DHM recipients' mothers were unable to breastfeed at all.

Raw data were collected in Microsoft Excel and analysed in SPSS; *z*‐scores were derived using the WHO Anthro Macro designed for R. Plots and regression analyses were also performed in R.

### 2.3 Ethical considerations

This study was assessed by the Imperial College London Research Ethics Committee as a service evaluation project and deemed exempt from formal ethics approval. All subjects' parents provided verbal or written consent for the recording of growth data for monitoring purposes by the milk bank after being made aware of the study aims and methodology.

## 3 RESULTS

Overall, 255 women donated their milk to the HMB between 2017 and March 2019. Thirty‐five infants were excluded due to missing growth information about the baby, 14 were excluded due to neonatal or infant deaths, four were excluded due to missing demographic data about the baby and two were excluded due to missing demographic data about the mother. Two hundred babies were ultimately included in the MOM cohort. Of the 49 infants receiving DHM in the community from the HMB between 2017 and 2019, the parents of 31 infants accepted invitations to be included in the study. Overall, 14 infants received DHM as a supplement to other feeds (MOM and/or IF), and 17 were exclusively fed DHM. Clinical and sociodemographic characteristics of the three cohorts are shown in Table 1. A greater proportion of women were multiparous in the exclusive MOM and supplemental DHM groups (46% and 50%) than in the exclusive DHM group (18%). There were high rates of university educated mothers in both DHM recipient groups (94% for exclusive and 79% for supplemental DHM infants), and the majority of women were living with their partners (94% for exclusive and 86% for supplemental DHM infants). Employment rates were higher in the MOM recipient group (89%) than in the exclusive and supplemental DHM groups (76% and 64%, respectively).

**TABLE 1:** Neonatal and maternal characteristics of participants

	Exclusive MOM (*n* = 201)	Exclusive DHM (*n* = 17)	Supplemental DHM (*n* = 14)
**Neonatal baseline characteristics**			
Type of birth			
Elective caesarian section (*n*, %)	NA	1 (6)	1 (7)
Emergency caesarian section (*n*, %)	NA	5 (29)	1 (7)
Vaginal delivery (*n*, %)	NA	11 (65)	12 (86)
GA at birth (mean, SD; week)	37.51 (4.68)	39.2 (3.17)	40 (2.16)
Premature (<37 weeks; *n*, %)	43 (22)	3 (18)	1 (7)
Birthweight (kg; mean, SD)	3.01 (0.952)	3.38 (0.82)	3.3 (0.76)
Female sex (*n*, %)	NA	6 (43)	6 (35)
Birth date (month/year; range)	05/2016–01/2019	08/2017–07/2019	07/2017–08/2019
Multiple gestation (*n*, %)	8 (4)	0 (0)	0 (0)
Antenatal corticosteroid exposure (*n*, %)	NA	3 (18)	1 (7)
Length of hospital stay (mean, SD; day)	NA	4 (7.45)	4 (5.75)
Neonatal unit stay (*n*, %)	58 (29)	4 (24)	3 (21)
**Maternal baseline characteristics**			
Pre‐pregnancy BMI, mean (SD)	NA	24.3 (5.15)	23 (2.25)
Serious illnesses during pregnancy (*n*, %)	NA	9 (53)	8 (57)
Antenatal smoking (*n*, %)	0 (0)	0 (0)	0 (0)
Multiparous (*n*, %)	92 (46)	3 (18)	7 (50)
**Family socioeconomic characteristics**			
Maternal age (year; mean, SD)	32.15 (4.32)	33.8 (4.53)	35 (6.03)
University educated (*n*, %)	NA	16 (94)	11 (79)
Employed (*n*, %)	177 (89)	13 (76)	9 (64)
Living with partner (*n*, %)	NA	16 (94)	12 (86)
Married (*n*, %)	NA	12 (71)	7 (50)

Abbreviations: BMI, body mass index; DHM, donor human milk; GA, gestational age; MOM, mother's own milk; NA, not applicable; SD, standard deviation.

Some characteristics differed between the exclusive DHM and supplemental DHM groups (Table 2). Infant weight loss (*n* = 6, 43%) and low maternal supply (*n* = 6, 43%) were common indications to receive supplemental DHM but were not an indication for exclusive DHM. The most common reasons for receiving exclusive DHM were maternal chemotherapy (*n* = 7, 41%) and bilateral mastectomy (*n* = 7, 41%). Infants in the exclusive DHM group were more likely to receive DHM at birth (77% vs. 7% of supplemental DHM recipients) and received greater volumes than infants receiving supplemental DHM.

**TABLE 2:** DHM characteristics of the DHM recipient groups

	Exclusive DHM (*n* = 17)	Supplemental DHM (*n* = 14)
Indication for receiving DHM		
Infant weight loss (*n*, %)	0 (0)	6 (43)
Maternal chemotherapy (*n*, %)	7 (41)	1 (7)
Bilateral mastectomy (*n*, %)	7 (41)	0 (0)
Low maternal supply (*n*, %)	0 (0)	6 (43)
Surrogacy (*n*, %)	1 (6)	0 (0)
Other maternal condition (*n*, %)	2 (12)	1 (7)
Babies receiving DHM at birth (*n*, %)	13 (77)	1 (7)
Age of baby at first DHM feed if not at birth (range, day)	2–105	2–119
Exclusive HMB DHM (*n*, %)	9 (53)	9 (64)
Volume of HMB DHM received per baby (mean, SD; L)	32.25 (19.1)	5.31 (5.98)
Duration of DHM (mean, SD; week)	8.59 (6.21)	6.18 (7.18)

Abbreviations: DHM, donor human milk; HMB, Hearts Milk Bank; SD, standard deviation.

**FIGURE 1:**
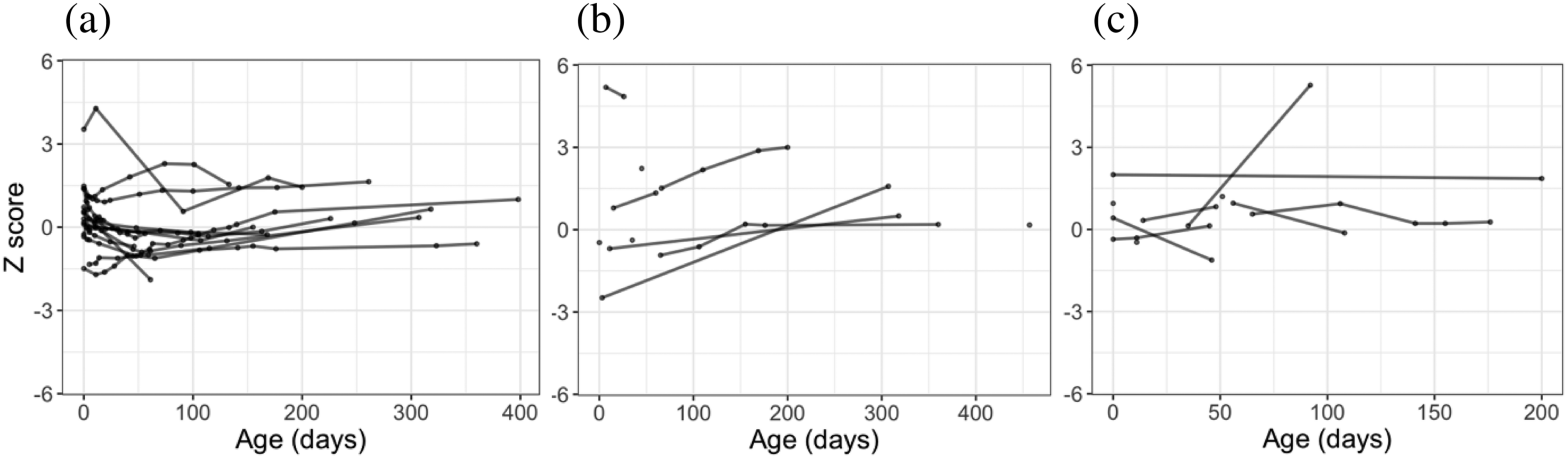
The *z*‐scores for (a) weight‐for‐age, (b) length‐for‐age, and (c) head circumference‐for‐age for each exclusive DHM infant

**FIGURE 2:**
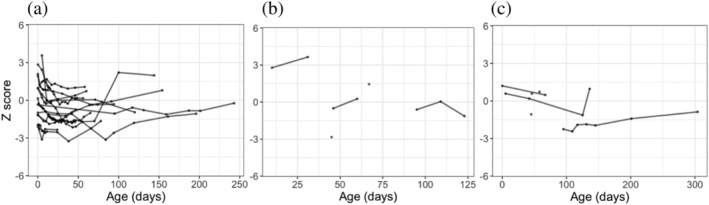
The *z*‐scores for (a) weight‐for‐age, (b) length‐for‐age, and (c) head circumference‐for‐age for each supplemental DHM infant

**FIGURE 3:**
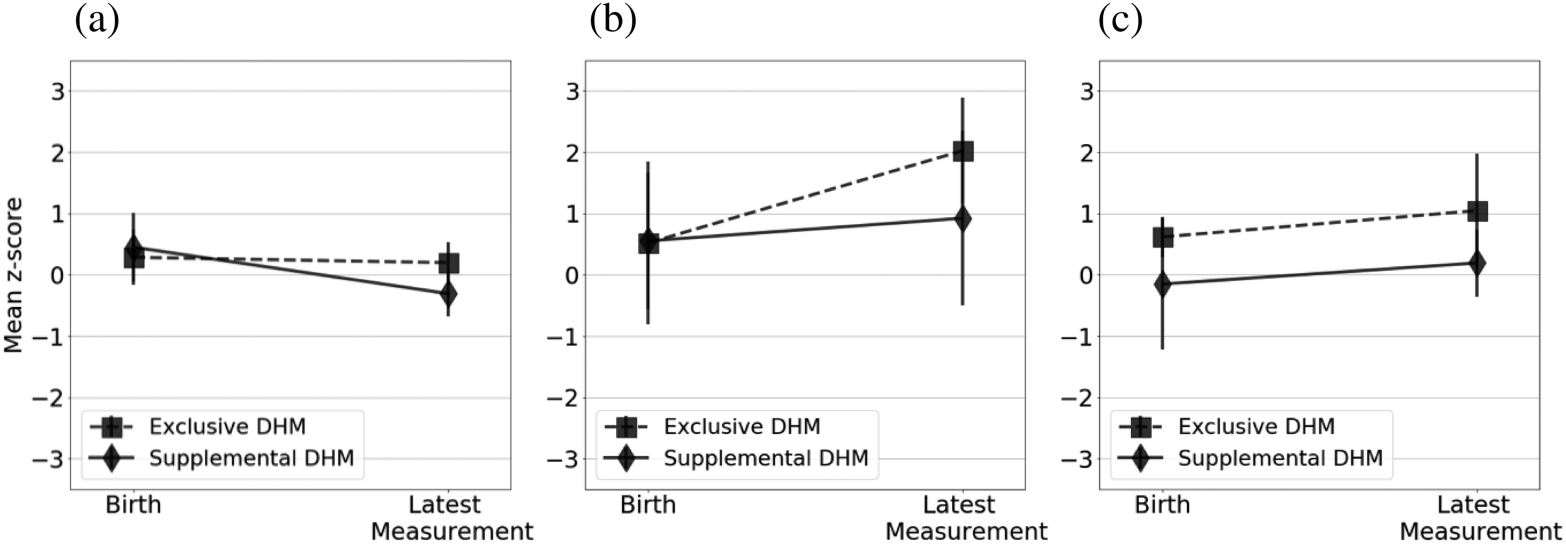
Mean *z*‐scores with standard error of the mean error bars for (a) weight‐for‐age, (b) length‐for‐age, and (c) head circumference‐for‐age at birth and last available measurement for each infant in the exclusive DHM and supplemental DHM groups

The *z*‐scores were derived for each anthropometric measurement recorded for each DHM infant. Table 3 shows the range of ages infants at their first and last DHM feed and at the last recorded measurement for weight, length and head circumference. The last measurements available for each baby were taken after the baby had stopped receiving DHM. For several babies, growth measurements were available before the baby had started receiving DHM. The trends in weight‐for‐age, length‐for‐age and head circumference‐for‐age *z*‐score from birth onwards were plotted for each individual infant according to whether they received exclusive and supplemental DHM (Figures 1 and 2, respectively). The overall trend for each DHM group was plotted in Figure 3. A multivariate regression model of *z*‐score against age was fitted with specific slopes for each DHM recipient group. The model could not find a statistically significant relationship between *z*‐score and age for each anthropometric measurement, suggesting no significant change in *z*‐score over the course of the observation period for all DHM recipients. When accounting for feeding type (exclusive DHM vs. supplemental DHM), the slopes remained nonsignificant, suggesting no specific feeding type association between *z*‐score and age. Overall, the model accounted for little of the variance in the data (adjusted *r* ^2^ = 9.5%).

**TABLE 3:** Age ranges for DHM recipients at first DHM feed, last DHM feed and last growth measurements

	Exclusive DHM recipients		Supplemental DHM recipients	
	All (*n* = 17)	Term (*n* = 14)	All (*n* = 14)	Term (*n* = 13)
Age at first DHM feed, day	0–105	0–105	0–119	0–115
Age at last DHM feed, day	21–175	21–175	9–185	9–185
Age at last weight measurement, day	46–398	46–398	24–243	24–243
Age at last length measurement, day	46–457	26–360	31–123	31–123
Age at last head circumference measurement, day	11–325	45–200	67–304	67–304

Abbreviations: DHM, donor human milk.

Growth velocity for each feeding group is shown in Table 4. Supplemental DHM term infants had the greatest growth velocity (11.65 g/kg/day, 95% CI 7.52–15.78 g/kg/day), followed by exclusive DHM infants (10.35 g/kg/day, 95% CI 8.32–12.37 g/kg/day) and MOM recipients (9.39 g/kg/day, 95% CI 8.67–10.11). A multivariate regression analysis adjusted for gestational age and birthweight found an overall significant association between weight and age, as expected. There was however no feeding type‐specific trend, suggesting that weight gain was not directly affected by feeding type. Again, only little of the variance in the data (*r* ^2^ = 8.5%) was accounted for by the model.

**TABLE 4:** Growth velocity (g/kg/day) and mean (95% CI) for each feeding group's full set and term infants

		Exclusive MOM	Exclusive DHM	Supplemental DHM
All infants	Population size (*n*)	200	17	14
	Growth velocity (g/kg/day; 95% CI)	9.39 (8.67–10.11)	10.35 (8.32–12.37)	11.65 (7.25–15.78)
Term infants	Population size (*n*)	152	14	13
	Growth velocity (g/kg/day; 95% CI)	8.08 (7.52–8.64)	9.49 (7.30–11.68)	12.00 (7.58–16.42)

Abbreviations: CI, confidence interval; DHM, donor human milk; MOM, mother's own milk.

Feeding tolerance for the DHM was investigated in the DHM recipient groups (Table 5). No parents reported serious adverse reactions that required hospitalization and none stopped giving DHM feeds due to poor tolerance. The majority of infants in both groups were bottle‐fed but a number of supplemental DHM recipients were fed using a Supplemental Nursing System, either on its own (*n* = 3, 21%) or in combination with bottles (*n* = 4, 29%), which consists of a container attached to a feeding tube that enters the baby's mouth during latching to the breast. Limited supply of DHM was a major reason DHM feeds were stopped in both groups (88% of exclusive DHM recipients and 43% of supplemental DHM recipients). In the supplemental DHM recipients, establishment of exclusive maternal supply (*n* = 5, 38%) and weaning onto solids (*n* = 3, 23%) was also common reasons. One family in the study stopped DHM feeds as a result of the impracticality of using DHM, specifically because other carers were not comfortable handling DHM. One infant in each group was still receiving DHM at the time of data collection.

**TABLE 5:** Mechanisms of DHM feeding and incidence of adverse reactions in the two DHM recipient groups

	Exclusive DHM recipients (*n* = 17)	Supplemental DHM recipients (*n* = 14)
DHM feeding mechanism		
Bottle, *n* (%)	17 (100)	7 (50)
Bottle and SNS, *n* (%)	0 (0)	4 (29)
SNS, *n* (%)	0 (0)	3 (21)
Adverse reactions		
Colic, *n* (%)	5 (29)	2 (14)
Fussiness, *n* (%)	0 (0)	1 (7)
Cramps, *n* (%)	1 (6)	1 (7)
Regurgitation, *n* (%)	8 (47)	6 (43)
Skin rashes, *n* (%)	1 (6)	0 (0)
Wheezing/sneezing, *n* (%)	0 (0)	0 (0)
Reasons for stopping DHM feeds		
Limited HMB supply, *n* (%)	15 (88)	6 (43)
Enough maternal supply, *n* (%)	NA	5 (36)
Weaning onto solids, *n* (%)	0 (0)	2 (14.3)
Adverse reaction to DHM, *n* (%)	0 (0)	0 (0)
Impracticality of DHM, *n* (%)	1 (6)	0 (0)

Abbreviations: DHM, donor human milk; HMB, Hearts Milk Bank; NA, not applicable; SNS, Supplemental Nursing System.

Of the infants belonging to the supplemental DHM cohort, 38% (5/13 infants) exclusively breastfed after receiving DHM. After excluding infants whose mothers would not be able to establish exclusive breastfeeding as a result of medical reasons (insufficient glandular tissue [*n* = 2], other maternal condition [*n* = 2] and chemotherapy [*n* = 1]), 63% (5/8 infants) were exclusively breastfed.

## 4 DISCUSSION

This study retrospectively evaluated the growth patterns of infants in the community who had received DHM exclusively for at least 3 weeks or to supplement their regular feeds. The multivariate regression analysis suggested no feeding method specific association between *z*‐score and age, and weight and age. These are tentative conclusions considering the low *r* ^2^ value of the model (9.5%), along with the study's small population size (both number of measurements and number of babies), so differences beyond this study cannot be ruled out. DHM was well‐tolerated, with no serious adverse reactions reported and no DHM feeds stopped due to poor feeding tolerance. Over half of women able to breastfeed (no risk of medication transfer or disease transmission, and enough breast tissue), established exclusive breastfeeding after supplementing their infant's feeds with DHM.

The use of DHM in preterm infants can reduce the risk and severity of necrotizing enterocolitis (M. Quigley, Embleton, & McGuire, 2018; M. Quigley & McGuire, 2014; M. A. Quigley, Henderson, Anthony, & McGuire, 2007), bronchopulmonary dysplasia (Villamor‐Martínez et al., 2018) and retinopathy of prematurity (Zhou, Shukla, John, & Chen, 2015). DHM also shortens the length of hospital stays compared with IF (Renfrew et al., 2009), which confers significant economic gains (Mahon, Claxton, & Wood, 2016). DHM is largely rationed for babies born <30 weeks gestation and is only available in 70% of hospitals in the United Kingdom (Battersby, Marciano Alves Mousinho, Longford et al., 2018).

Increasing public awareness of milk bank services has increased demand for access to DHM by parents, and in‐hospital use is increasing globally (PATH, 2019; Shenker, 2020). However, there remains a paucity of literature regarding the use and growth outcomes of DHM in nonhospitalized infants, particularly term infants. Limited studies exist regarding DHM use in term infants (Belfort et al., 2018; Ferrarello, Schumacher, & Anca, 2019; Kair, Colaizy, Hubbard, & Flaherman, 2014; Kair & Flaherman, 2017; Kair, Flaherman, & Colaizy, 2019; Sen et al., 2018) in addition to limited numbers of case series (Reimers, Shenker, Weaver, & Coutsoudis, 2018; Tully, Lockhart‐Borman, & Updegrove, 2004), but contrary to the present study, all these infants were hospitalized or ill, and none listed growth as a primary outcome.

Despite the scant available evidence, the American Academy of Paediatrics recommends DHM over IF when a feeding supplement to MOM is medically indicated (AAP, Breastfeeding Section, 2012). This current study provides preliminary evidence in support of this recommendation by showing that the growth velocity of infants was not negatively affected by receiving DHM. Our study is also unique in studying a group of infants who were exclusively fed DHM for a prolonged period (>3 weeks). There are concerns that the processing of DHM affects its nutritional content and therefore would not support infant growth. Pasteurization of the DHM eradicates bile salt‐stimulated lipase activity (Peila et al., 2016), an enzyme required for fat absorption, and leads to reductions in some metabolic hormones such as adiponectin (Ley, Hanley, Stone, & O'Connor, 2011), whereas growth factors, human milk oligosaccharides and cytokine levels are unchanged (Bertino et al., 2008; Coscia et al., 2015; Peila et al., 2016). Some components of human milk vary with infant age and environment (Ballard & Morrow, 2013), and with gestational maturity of the baby (Bokor, Koletzko, & Decsi, 2007; Gila‐Diaz et al., 2019; Granot, Ishay‐Gigi, Malaach, & Flidel‐Rimon, 2016; Sabatier et al., 2019; Xavier et al., 2018), and each of these aspects will need to be considered in future functional profiling of DHM.

The feeding tolerance for DHM was also analysed. This has previously only been studied in preterm infants who have much more immature gastrointestinal systems than term infants (Costa et al., 2018). Studies that examine in‐hospital infant feeding measure feeding tolerance by assessing the infant's age when full enteral feeding is achieved. As this study focused on infant feeding outside of the hospital, feeding tolerance was assessed through the incidence of key gastrointestinal symptoms that are reported in trials comparing infant feeding strategies (US Department of Health and Human Services, 2019). A similar number of babies in each DHM group were reported by their mothers to have these symptoms. Moreover, no infants stopped receiving DHM feeds due to an adverse reaction, together suggesting that DHM was well‐tolerated.

The WHO recommends that infants should be exclusively breastfed for 6 months, but this target is currently only met by 38% of infants worldwide (United Nations Children's Fund, 2018). Breastfeeding rates vary significantly between countries, with rates particularly low in the United Kingdom, where only 0.5% of children are breastfed at 12 months (Victora et al., 2016). When used inappropriately, DHM can have a negative impact on the likelihood of establishing maternal breastfeeding (Chantry, Dewey, Peerson, Wagner, & Nommsen‐Rivers, 2014; Loughlin, Clapp‐Channing, Gehlbach, Pollard, & McCutchen, 1985; Williams, Nair, Simpson, & Embleton, 2016), possibly through mothers underestimating the importance of their own breast milk and reducing the likelihood of infants having sufficient opportunities to latch and suckle. The latter can be prevented by giving small volumes of DHM at the end of feeds or by using a Supplemental Nursing System. Other studies have noted improvements in breastfeeding with DHM use, most notably a 2‐year audit of 22 newborn intensive care units in the United States that reflected a 10% median increase in average discharge breastfeeding rate (Kantorowska et al., 2016) and an Italian study that identified significantly higher breastfeeding rates at discharge in units that provided DHM (29.6%) than units that did not (16.0%) (Arslanoglu et al., 2013). No studies have investigated the effect of supplemental DHM feeds in the community on breastfeeding rates. The present study focused on rates of exclusive breastfeeding after DHM supplementation. When accounting for the breastfeeding of some mothers being limited physiologically, 63% of infants in the supplemental DHM group went on to be exclusively breastfed after DHM. These exclusive breastfeeding rates are promising in light of the UK's very low breastfeeding rates, and the increasing recognition that early difficulties predict the likelihood of breastfeeding cessation (Odom, Li, Scanlon, Perrine, & Grummer‐Strawn, 2013).

The main limitations of this study are due to its observational retrospective design. There was no consistency in the equipment used or the age of the infants when the anthropometric measurements were taken, as the growth of healthy infants in the United Kingdom is measured at home by health visitors. Occasionally, it is not possible to gain growth measurements at the point in time that DHM supply ceased. In this instance, the closest recorded measurement in the infant's health records were used for the analysis. However, the findings of positive growth rates during the period of time DHM was used were supported by parent interviews during the qualitative aspect of the study (data not shown), with adequate growth reported in each individual infant's case. There was also a large variation in the number of measurements available per infant, due to length and head circumference of healthy infants not being routinely measured. The number of study subjects was small since the HMB became functional 2 years ago and has thus provided DHM to only a limited number of infants in the community. Feeding tolerance data were not available about the MOM recipients, which limits our understanding of the incidence of gastrointestinal symptoms with DHM in comparison to MOM.

## 5 CONCLUSION

This study was, to the best of our knowledge, the first to investigate growth in nonhospitalized infants fed DHM exclusively or as a feeding supplement. The results showed that these infants grew appropriately and that the DHM was well‐tolerated. Future prospective studies, including randomized controlled trials with larger sample sizes and accurate infant measurement criteria, will be needed to support these findings, as well as to determine the impact of DHM availability on maternal psychology and breastfeeding rates.

## CONFLICTS OF INTEREST

Natalie Shenker and Gillian Weaver are nonremunerated cofounders of the Human Milk Foundation and the Hearts Milk Bank. The other authors have no potential conflicts of interest to disclose. R. B. reports receiving personal fees for consultancy in the past 3 years from Prota Therapeutics and DBV Technologies who develop food allergy treatments and from the Dairy Goat Cooperative for design of a clinical trial. Imperial College London has a formal research and innovation partnership with Nestle. None of the authors or their research groups are involved in this work in any way.

## CONTRIBUTIONS

SB collected data, carried out the analyses, drafted the initial manuscript, and reviewed and revised the manuscript. RB supervised the data collection and critically reviewed the manuscript. GW co‐designed the study and reviewed and revised the manuscript. NS conceptualized and designed the study, coordinated and supervised the data collection, assisted with data analysis, and reviewed and revised the manuscript. All authors approved the final manuscript as submitted and agreed to be accountable for all aspects of the work.
